# Supplementary material for: Data‐driven modeling reconciles kinetics of ERK phosphorylation, localization, and activity states
Source: Mol Syst Biol. 2014 Jan 31;10(1):718. doi: 10.1002/msb.134708 (PMC4023404; doi:10.1002/msb.134708)
Supplement: Supplementary file 1 — Supplementary Legends [file MSB-10-1-718-s046.pdf]

SUPPLEMENTARY FIGURE LEGENDS

FIGURE S1. **ERK phosphorylation kinetics assessed by immunoblotting.** NIH 3T3 cells were either left unstimulated or stimulated with the indicated concentrations of PDGF for various times (5, 15, 30, 60, and 120 minutes). Phosphorylated ERK (p-ERK) and total ERK (t-ERK) were assessed by quantitative immunoblotting in three independent experiments. (A) Representative blots. (B&C) Separate, normalized quantification of ERK1 (B) and ERK2 (C) phosphorylation. Values are reported as mean ± s.e.m. in arbitrary units (n = 3).

FIGURE S2. **Quantification of mono-phosphorylated ERK2 states by LC/MS/MS analysis using Skyline software.** The top row presents label-free quantitative LC/MS/MS results, analyzed using Skyline software, showing kinetics of mono-phosphorylated (pT181 and pY183) and dephosphorylated (ppERK2) forms of ERK2 in NIH 3T3 cells, maximally stimulated with PDGF for the indicated times. The bottom row shows the homologous phosphorylation states of ERK1. Values are normalized and reported as mean ± s.e.m. in arbitrary units (n = 3).

FIGURE S3. **Validation of the EKAR biosensor.** Nuclear ERK activity was measured by ratio imaging of nuclear EKAR FRET signal ratio in NIH 3T3 cells. Pretreatment with an inhibitor of MEK (A, n = 7) or of ERK (B, n = 9) blocks the EKAR response elicited by maximal PDGF stimulation. The results are reported as mean (black line) ± 95% confidence interval (gray).

FIGURE S4. **Individual cell responses measured by mCherry-ERK2 localization and EKAR biosensors.** Kinetic traces for individual cells (gray) are overlaid with the mean kinetics reported in Fig. 2 of the paper (red). A-D correspond to the results shown in Fig. 2 panels B, C, E, and F respectively.

FIGURE S5. **Nuclear ERK dynamics elicited by FGF-2 stimulation.** NIH 3T3 cells cotransfected with mCherry-ERK2 and nuclear EKAR were stimulated with 1 nM FGF-2 rather than PDGF. Mean nuclear translocation (n = 8) and nuclear EKAR (n = 10; i.e., 8 of these also showed suitable expression of mCherry-ERK2) kinetics were assessed by live-cell microscopy. In each of these plots, the gray regions report 95% confidence intervals.

FIGURE S6. **Phosphorylation time course of MEK1 Thr292, a cytosolic substrate of ERK.** *Left:* Representative blots showing phosphorylation of MEK1 Thr292 (p-Thr292) and total MEK1 (t-MEK1) levels as a function of time following maximal PDGF stimulation of NIH 3T3 cells. *Right:* Quantification showing the normalized means in arbitrary units of two independent experiments. Each error bar reports the range of the two normalized values.

FIGURE S7. **A kinetic model lacking substrate interactions fails to reconcile observed ERK phosphorylation, localization, and activity responses.** (A) Schematic of a model lacking substrate interactions. (B-J) Alignment of this model to data as performed for the substrate model shown in Fig. 4 B-J, respectively. Note in particular that the ensemble-averaged model fit in this case generates time courses that, unlike the experimental data, are similar in shape for whole-cell ERK phosphorylation (C), ERK nuclear translocation (F), and ERK nuclear activity (H). For those outputs, the ensemble fit shows little variation, indicating that this model is consistently constrained to strike a compromise between the high degree of adaptation seen in

the ERK phosphorylation and nuclear translocation responses versus the low degree of adaptation seen in the nuclear activity kinetics.

FIGURE S8. **A kinetic model lacking substrate interactions but including two-step nuclear translocation of ERK fails to reconcile observed ERK phosphorylation, localization, and activity responses.** (A) Schematic of a model lacking substrate interactions but including the release from a shuttling protein (representing importin-7) as an additional step preceding the delivery of active ERK to the nucleus. (B-J) Alignment of this model to data as performed for the substrate model shown in Fig. 4 B-J, respectively. Like the model fit shown in Fig. S7, this one suffers from the same qualitative deviations from the experimental data, and consistently so across the ensemble of parameter sets.

FIGURE S9. **Adding two-step nuclear translocation of ERK to the model with substrate interactions does not discernibly improve the fit.** (A) Schematic of a model that includes both substrate interactions (as in Fig. 4) and release from a shuttling protein in the nucleus (as in Fig. S6). (B-J) Alignment of this model for comparison to the substrate model shown in Fig. 4 B-J, respectively.

FIGURE S10. **Comparison of the nuclear Elk-1 phosphorylation time course to modeling results.** The shape of the free, active ERK in the nucleus (n_{pp}, red) and the stoichiometry of nuclear substrate phosphorylation (p_n/s_{nT}, blue) from the ensemble model fit (mean ± s.d., $n = 10^4$) are plotted along with the nuclear Elk-1 phosphorylation data from Fig. 2G (black). The phospho-Elk-1 data and the n_{pp} curve were rescaled to set their maximum values equal to one.

FIGURE S11. **Transient buffering by substrates is sufficient for adaptation of ERK phosphorylation and nuclear translocation.** (A) Schematic of a model lacking negative feedback. The level of active MEK is taken as a constant for $t > 0$. (B-I) Alignment of this model to data as performed for the substrate model shown in Fig. 4 C-J, respectively.

FIGURE S12. **Nuclear localization of ERK2 at sequential PDGF doses.** (A) ERK2 nuclear localization kinetics of individual cells sequentially stimulated with 30 pM and 1 nM PDGF (n = 8). Kinetic traces for individual cells (gray) are overlaid with the mean kinetics reported in Fig. 2 of the paper (red). Kinetic traces for individual cells (gray) are overlaid with the mean kinetics reported in Fig. 6C of the paper (red). (B) Control experiment in which the first stimulation period was replaced with the addition of buffer only. The top panel shows the average response, reported as mean (black line) ± 95% confidence interval (gray) (n = 8). The bottom panel shows the individual cell responses plotted as in A.
