# Supplementary figures and images for: Data‐driven modeling reconciles kinetics of ERK phosphorylation, localization, and activity states
Source: Mol Syst Biol. 2014 Jan 31;10(1):718. doi: 10.1002/msb.134708 (PMC4023404; doi:10.1002/msb.134708)

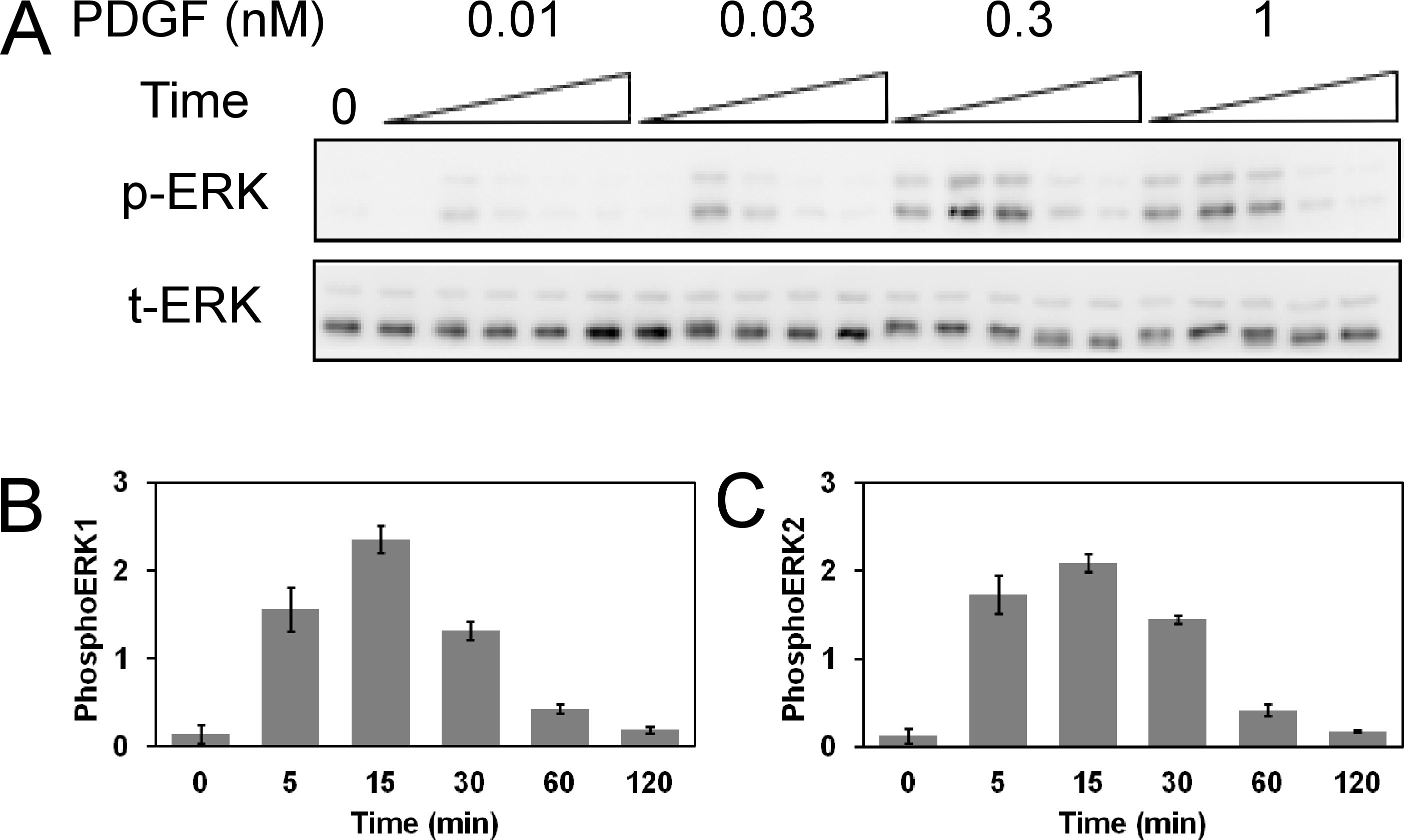

Supplement: Supplementary file 2 — Supplementary Figure 1 [file MSB-10-1-718-s048.tif]

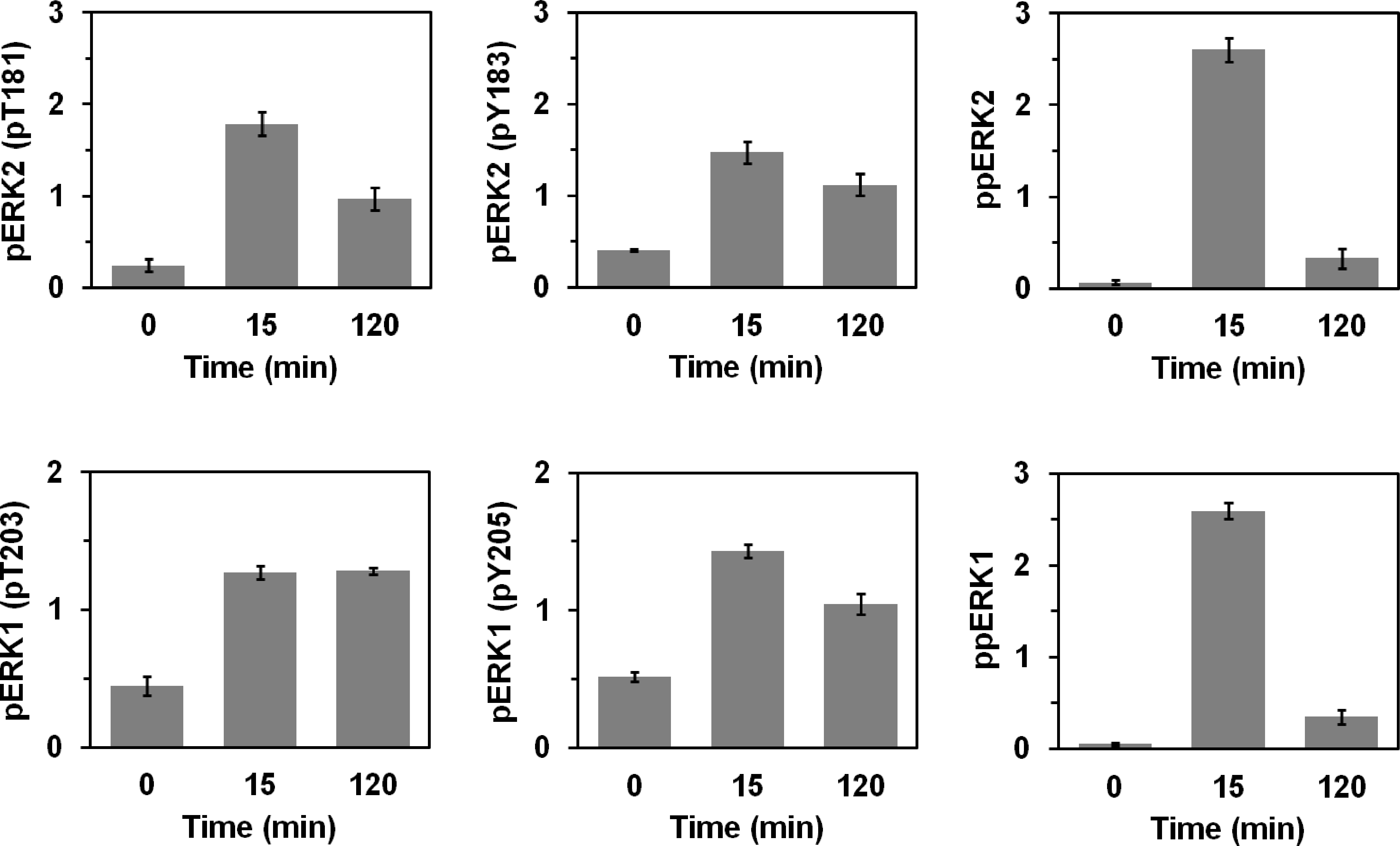

Supplement: Supplementary file 3 — Supplementary Figure 2 [file MSB-10-1-718-s049.tif]

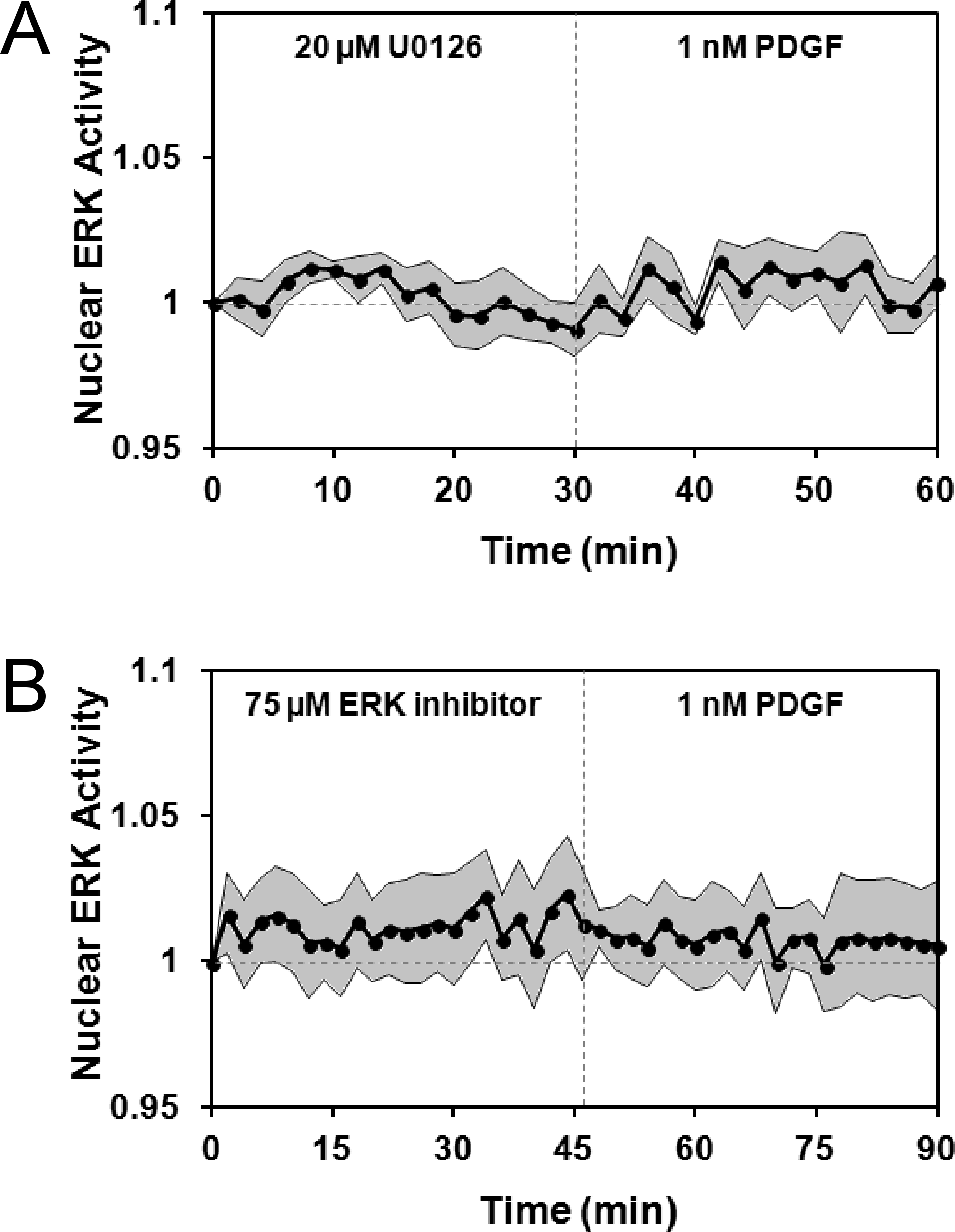

Supplement: Supplementary file 4 — Supplementary Figure 3 [file MSB-10-1-718-s050.tif]

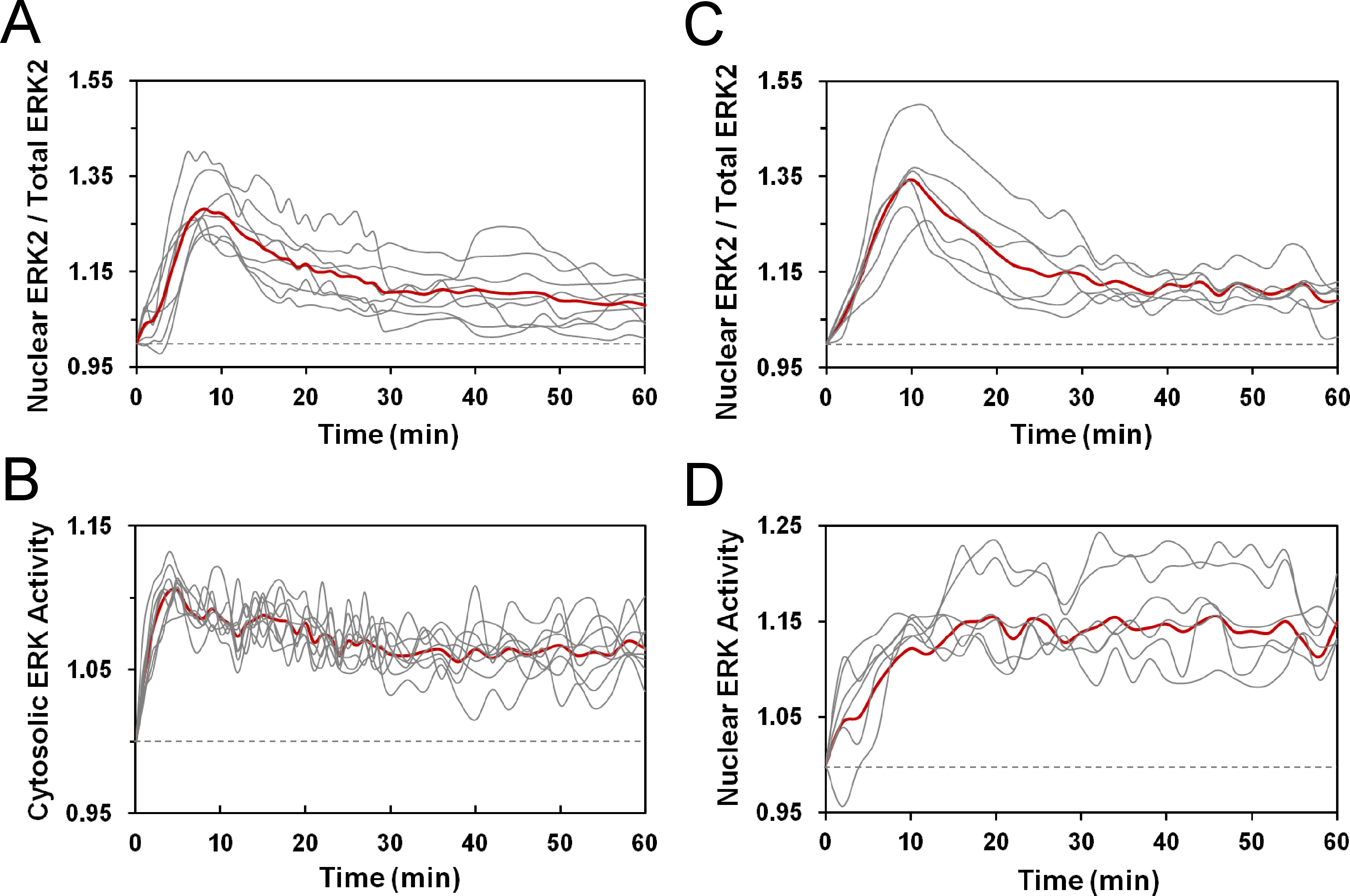

Supplement: Supplementary file 5 — Supplementary Figure 4 [file MSB-10-1-718-s051.tif]

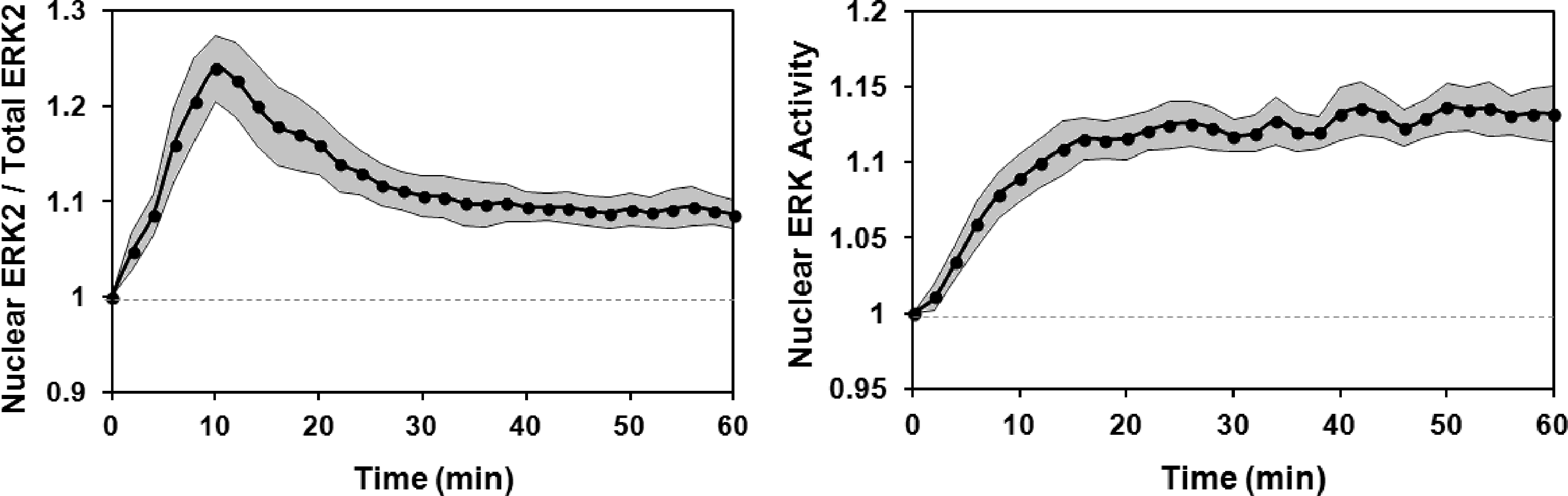

Supplement: Supplementary file 6 — Supplementary Figure 5 [file MSB-10-1-718-s052.tif]

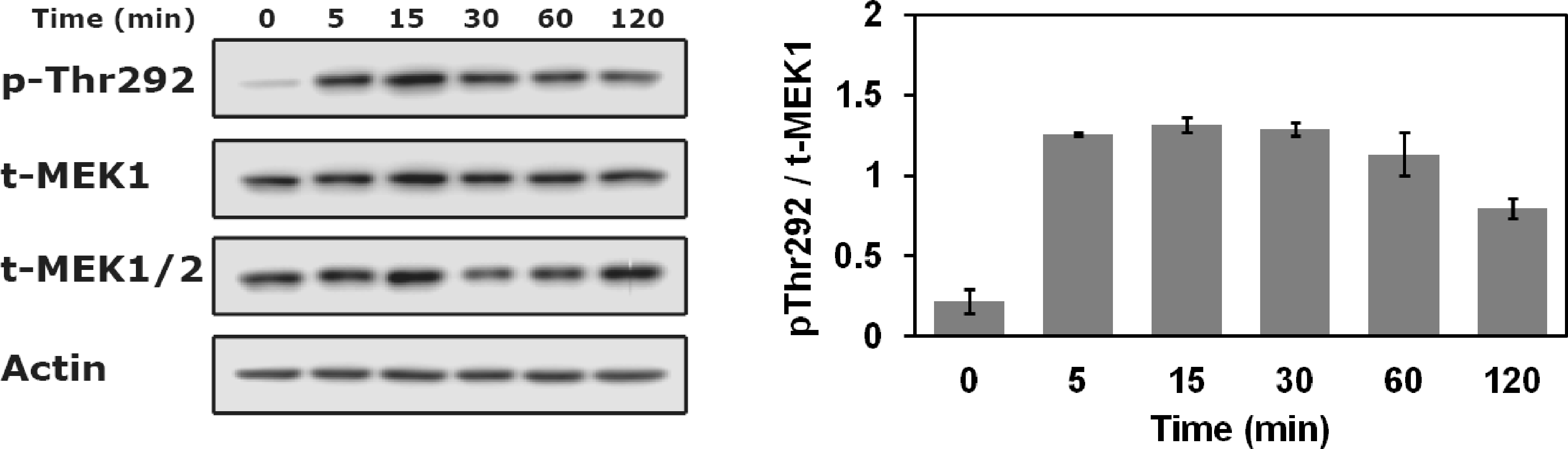

Supplement: Supplementary file 7 — Supplementary Figure 6 [file MSB-10-1-718-s053.tif]

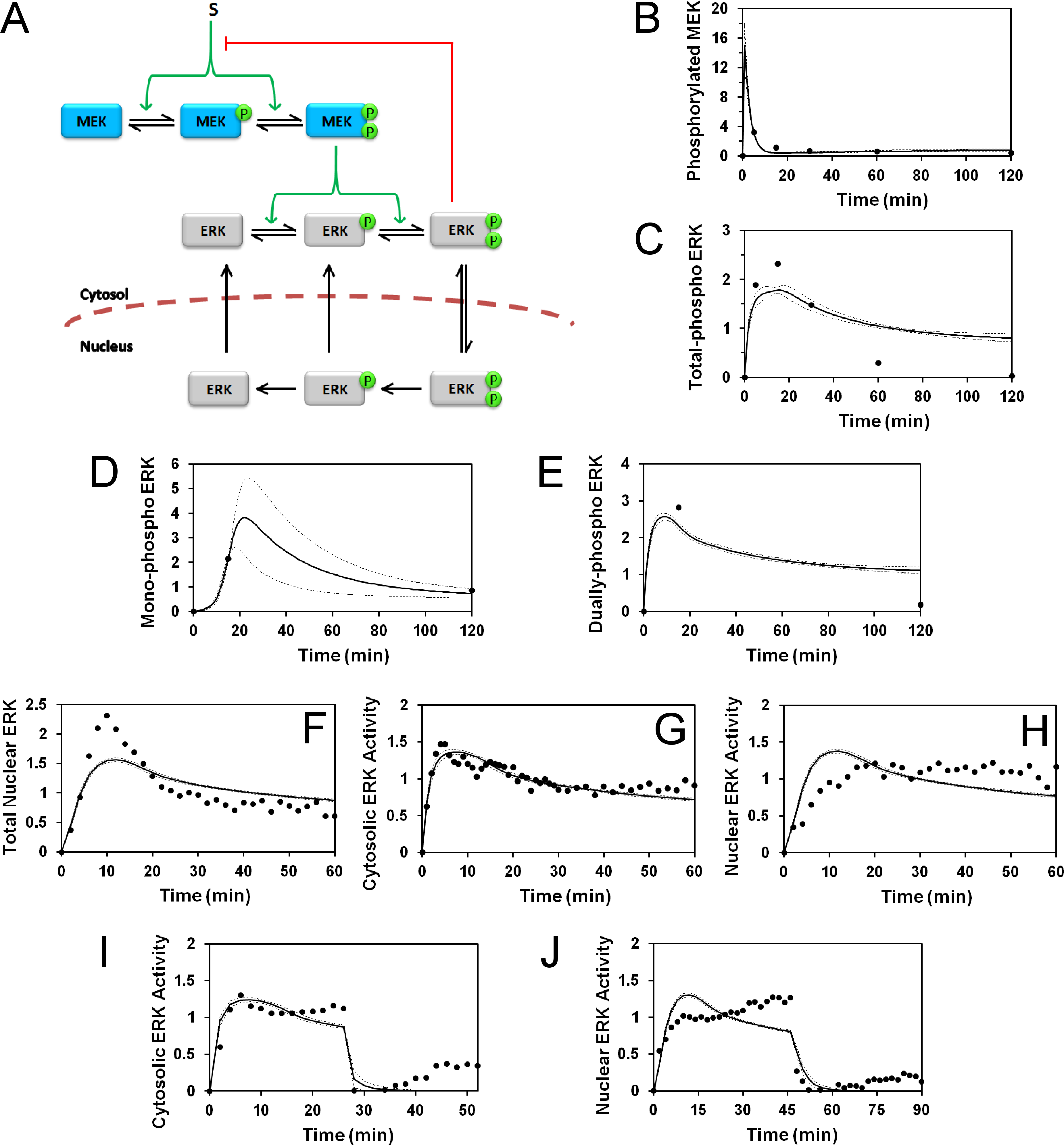

Supplement: Supplementary file 8 — Supplementary Figure 7 [file MSB-10-1-718-s054.tif]

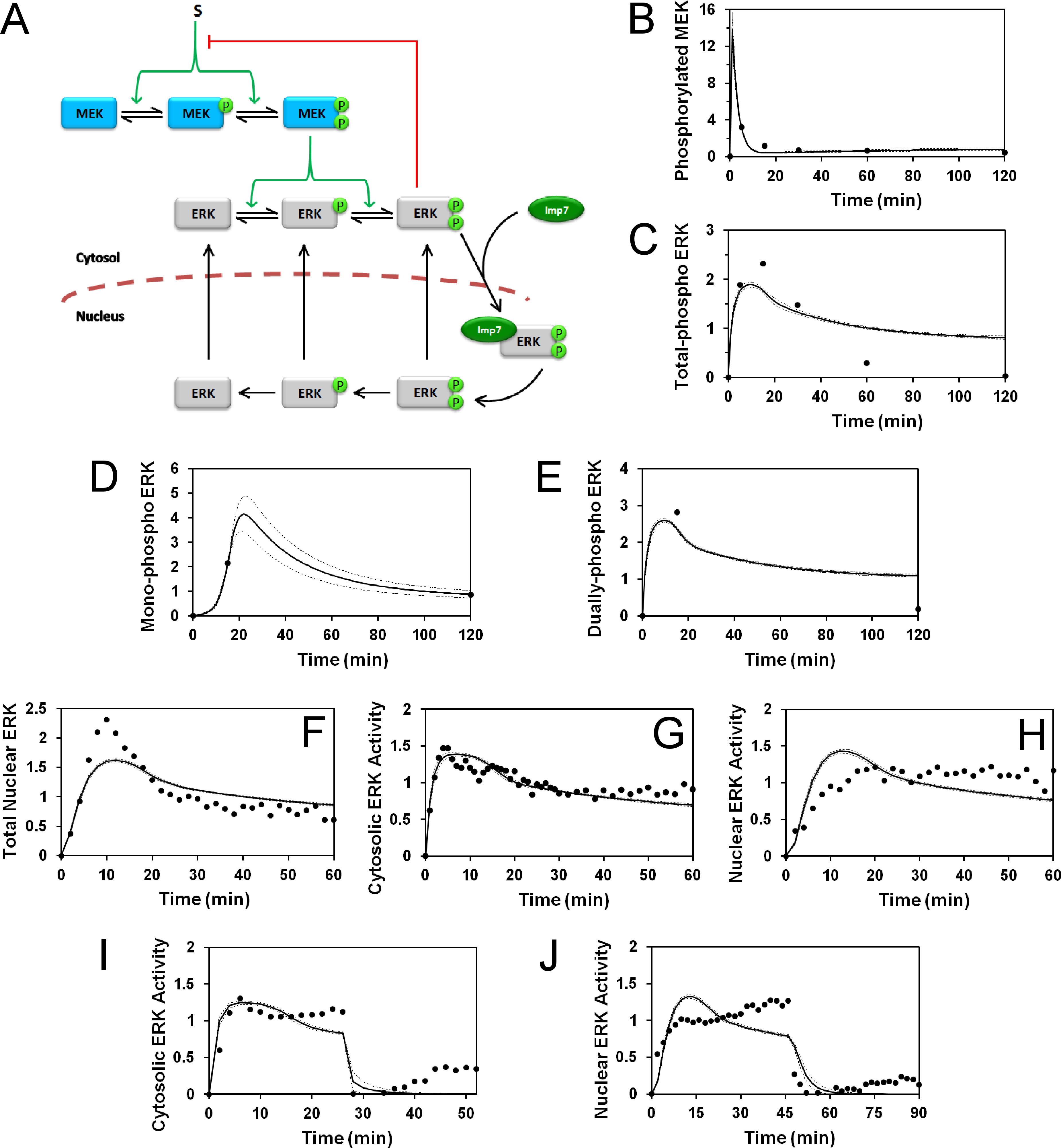

Supplement: Supplementary file 9 — Supplementary Figure 8 [file MSB-10-1-718-s055.tif]

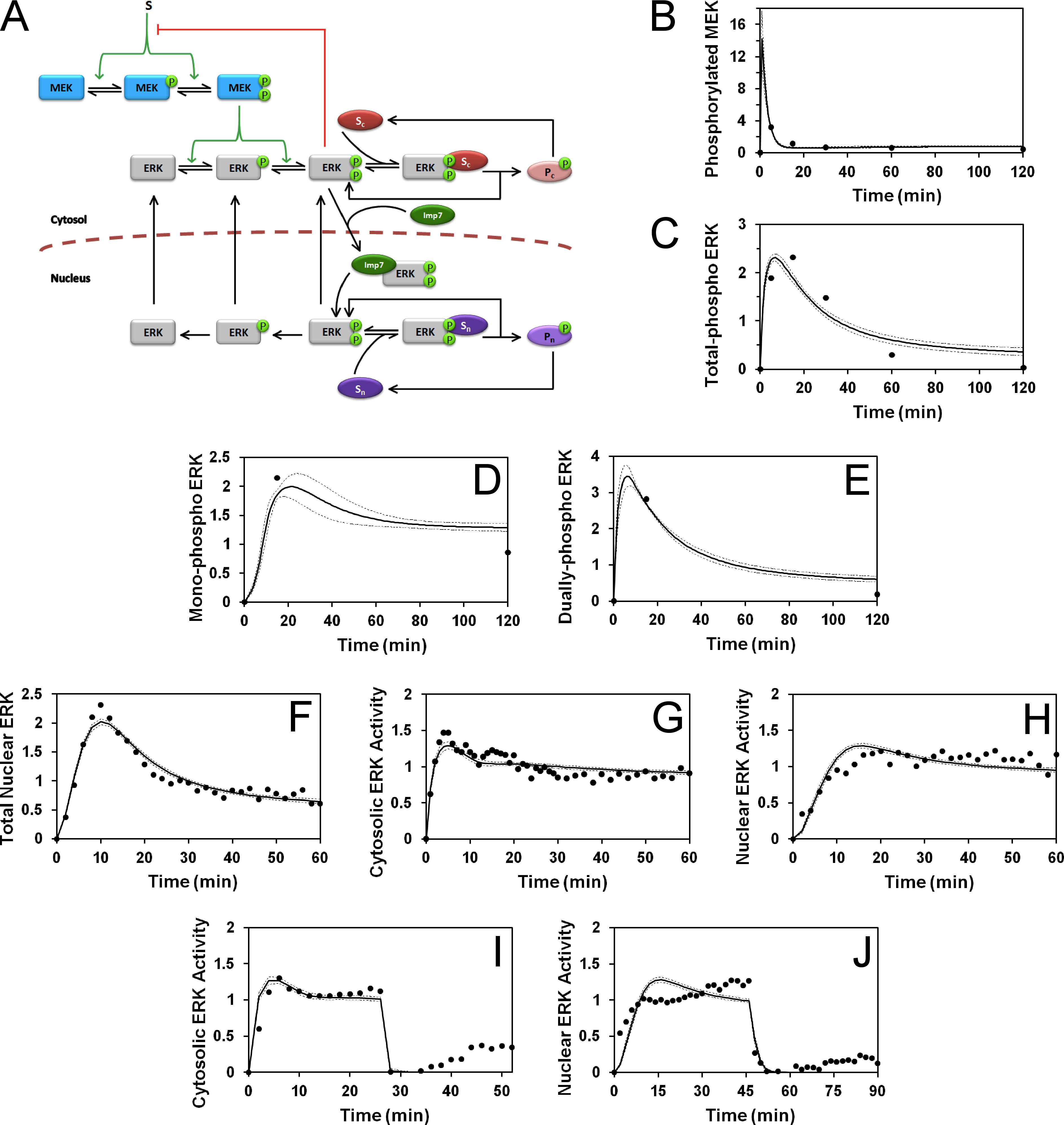

Supplement: Supplementary file 10 — Supplementary Figure 9 [file MSB-10-1-718-s056.tif]

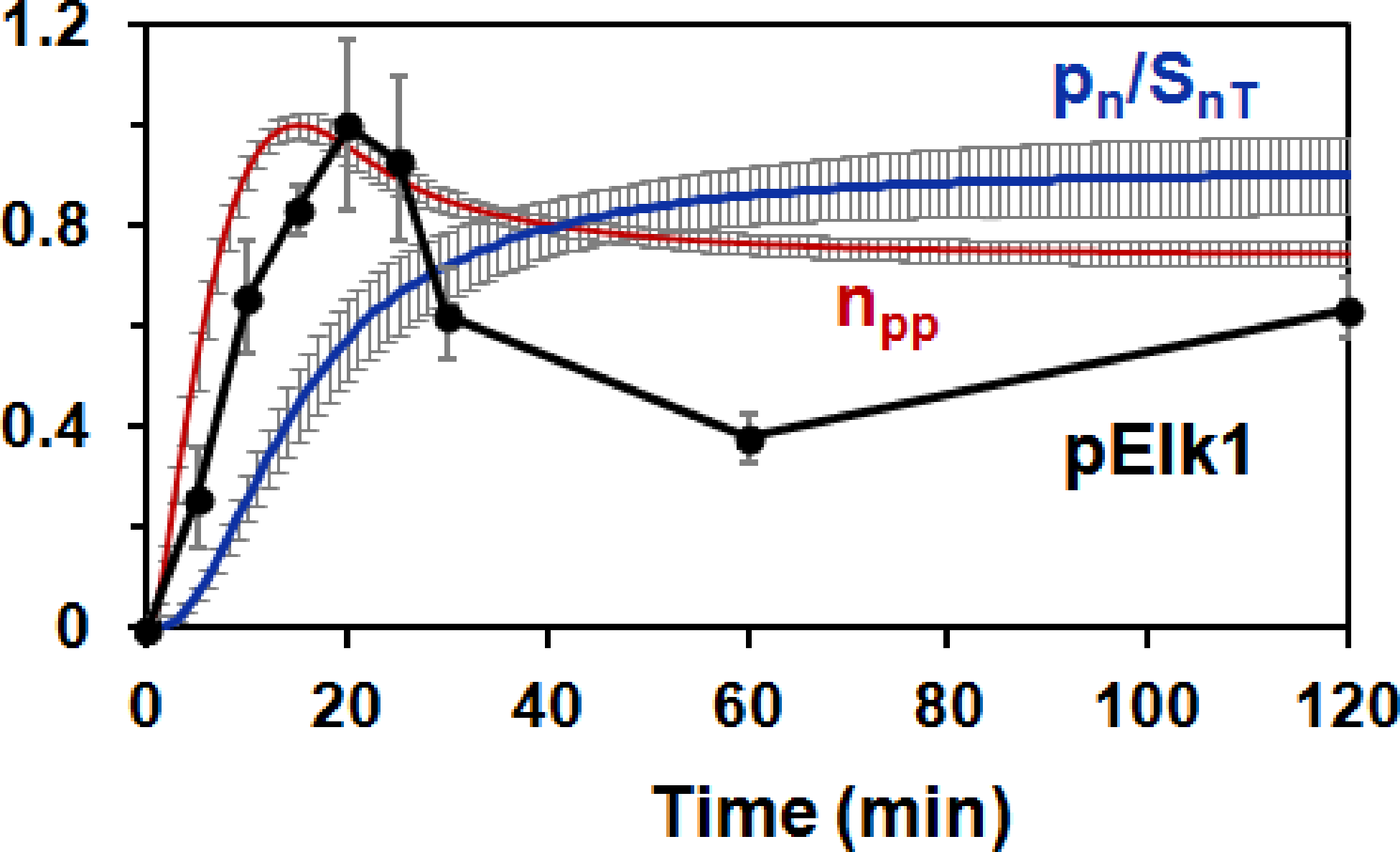

Supplement: Supplementary file 11 — Supplementary Figure 10 [file MSB-10-1-718-s057.tif]

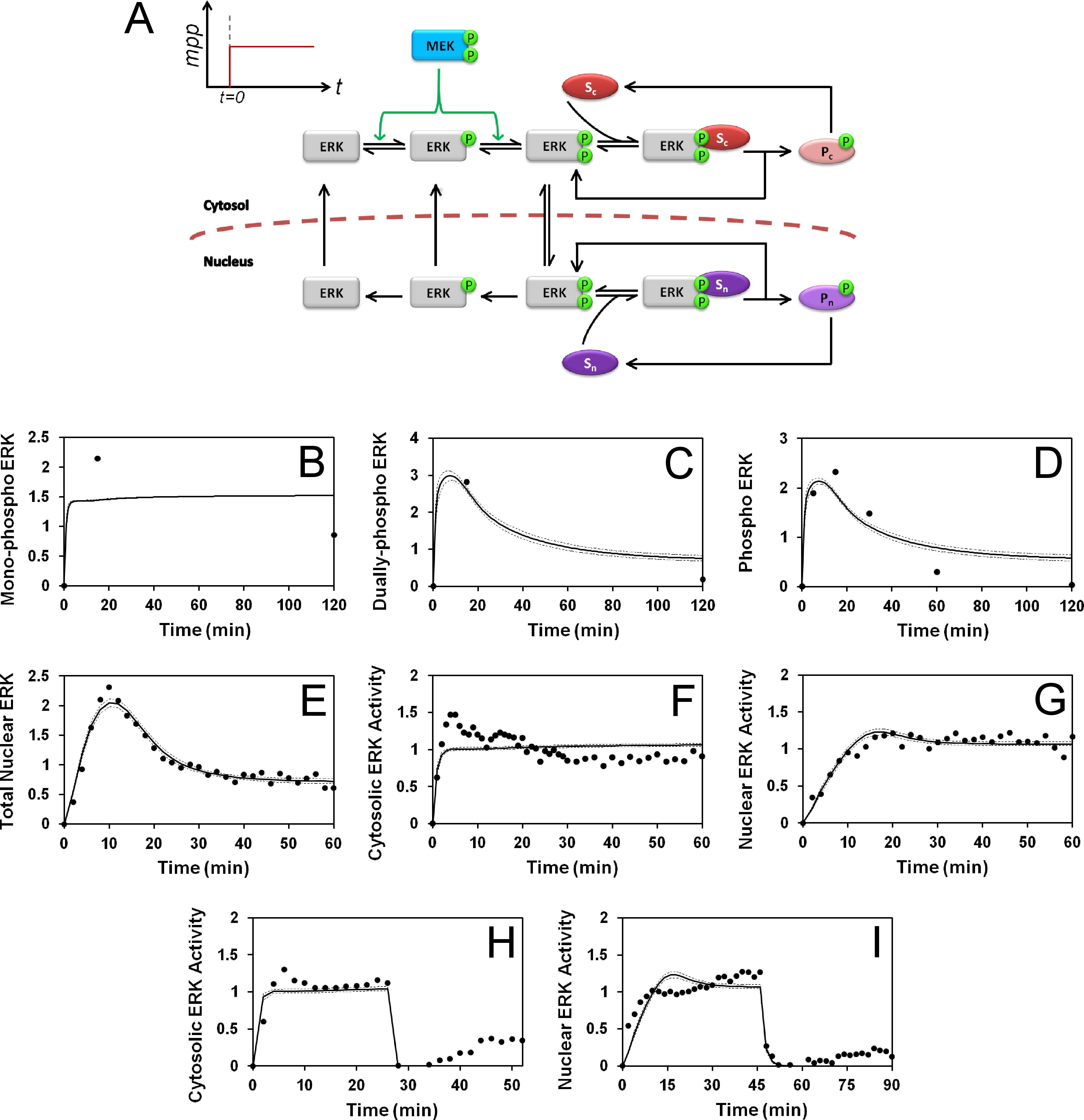

Supplement: Supplementary file 12 — Supplementary Figure 11 [file MSB-10-1-718-s058.tif]

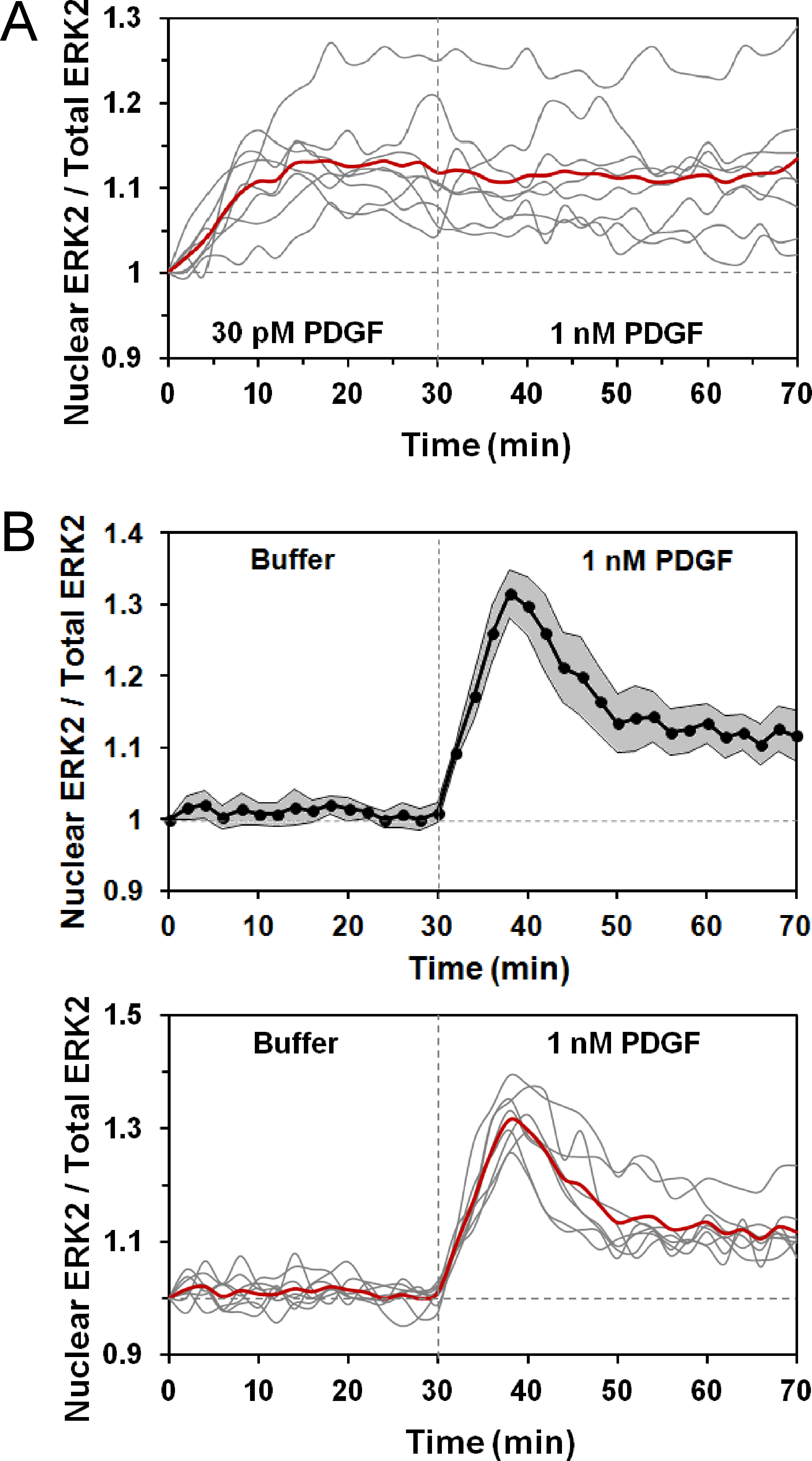

Supplement: Supplementary file 13 — Supplementary Figure 12 [file MSB-10-1-718-s059.tif]
