# Supplementary material for: Data‐driven modeling reconciles kinetics of ERK phosphorylation, localization, and activity states
Source: Mol Syst Biol. 2014 Jan 31;10(1):718. doi: 10.1002/msb.134708 (PMC4023404; doi:10.1002/msb.134708)
Supplement: Supplementary file 14 — Supplementary Reference [file MSB-10-1-718-s060.pdf]

References

Cirit M, Haugh JM (2012) Data-driven modelling of receptor tyrosine kinase signalling networks quantifies receptor-specific potencies of PI3K- and Ras-dependent ERK activation. *Biochem. J.* **441:** 77-85

Cirit M, Wang C-C, Haugh JM (2010) Systematic quantification of negative feedback mechanisms in the extracellular signal-regulated kinase (ERK) signaling network. *J. Biol. Chem.* **285:** 36736-36744

Hirashima T (2012) A kinetic model of ERK cyclic pathway on substrate control. *Math. Biosci.* **239:** 207-212

Horgan AM, Stork PJ (2003) Examining the mechanism of Erk nuclear translocation using green fluorescent protein. *Exp. Cell Res.* **285:** 208-220

Metropolis N, Rosenbluth A, Rosenbluth M, Teller A, Teller E (1953) Equation of state calculations by fast computing machines. *J Chem Phys* **21:** 1087-1092

Wang C-C, Cirit M, Haugh JM (2009) PI3K-dependent crosstalk interactions converge with Ras as quantifiable inputs integrated by Erk. *Mol. Syst. Biol.* **5:** article no. 246
