# Supplementary material for: Data‐driven modeling reconciles kinetics of ERK phosphorylation, localization, and activity states
Source: Mol Syst Biol. 2014 Jan 31;10(1):718. doi: 10.1002/msb.134708 (PMC4023404; doi:10.1002/msb.134708)
Supplement: Supplementary file 15 — Supplementary Table 1 [file MSB-10-1-718-s062.pdf]

Parameter	Definition	Min	Q1	Median	Q3	Max
k_1	Rate constant, MEK phosphorylation	0.0892	1.23	7.83	30.0	122
k_{-1}	Rate constant, pMEK dephosphorylation	0.168	0.716	2.40	23.3	124
k_2	Rate constant, pMEK phosphorylation	0.199	1.52	7.15	68.9	354
k_{-2}	Rate constant, ppMEK dephosphorylation	0.133	0.173	0.201	0.439	0.581
K_i	Saturation constant, MEK kinase desensitization	1.01e3	4.15e3	5.57e3	7.22e3	9.99e3
s_{cT}	Total substrate in cytosol	1.25e-4	0.0273	10.6	296	807
k_3	Rate constant, ERK phosphorylation, cytosol	0.146	0.520	1.09	1.80	6.39
k_{-3}	Rate constant, pERK dephosphorylation, cytosol	4.76e-3	0.0328	0.0487	0.0982	0.279
k_4	Rate constant, pERK phosphorylation, cytosol	1.03	5.92	23.5	2.52e3	9.68e3
k_{-4}	Rate constant, ppERK dephosphorylation, cytosol	1.15e-4	0.0437	1.18	121	327
k_n	Rate constant, ppERK nuclear import	0.217	1.07	2.14	4.46	10.1
k_{-n}	Rate constant, ERK nuclear export	0.436	0.797	0.956	1.21	6.94
$k_{on,c}$	Rate constant, ppERK-substrate association, cytosol	1.00e-4	1.47e-3	0.0169	16.6	50.8
$k_{off,c}$	Rate constant, ppERK-substrate dissociation, cytosol	6.37e-4	1.43	93.9	266	587
$k_{cat,c}$	Rate constant, substrate phosphorylation, cytosol	1.85e-4	0.317	9.38	21.6	62.3
k_{-pc}	Rate constant, product dephosphorylation, cytosol	1.01e-4	7.21e-4	4.38e-3	0.0390	3.69
s_{nT}	Total substrate in nucleus	0.257	6.31	90.78	212	5.23e3
k_{-5}	Rate constant, pERK dephosphorylation, nucleus	1.01e-4	0.0216	0.0648	0.907	2.93
k_{-6}	Rate constant, ppERK dephosphorylation, nucleus	1.20e-4	0.0440	1.60	3.34	8.29
$k_{on,n}$	Rate constant, ppERK-substrate association, nucleus	1.60	5.17	6.46	10.4	185
$k_{off,n}$	Rate constant, ppERK-substrate dissociation, nucleus	1.07e-4	0.0446	1.54	4.77	299
$k_{cat,n}$	Rate constant, substrate phosphorylation, nucleus	0.0969	4.38	30.8	54.9	1.32e3
k_{-pn}	Rate constant, product dephosphorylation, nucleus	1.03e-4	1.01e-3	2.54e-3	0.0111	0.0239
α_M	Mono-/di-phosphorylated MEK in Western blot signal	1.02e-4	3.93e-3	0.0187	0.114	0.371
α_E	Mono-/di-phosphorylated ERK in Western blot signal	1.01e-4	8.69e-3	0.0265	0.154	1.04

Table S1. Statistics of the parameter set ensemble for the 'substrate' model. All parameters labeled as rate constants have units of min^{-1}; all others are dimensionless. For each parameter, the minimum (Min), first quartile (Q1), median, third quartile (Q3), and maximum (Max) of the 10,000 values in the ensemble are listed. These values are also shown in box plot form on the following page.
