# Supplementary material for: Data‐driven modeling reconciles kinetics of ERK phosphorylation, localization, and activity states
Source: Mol Syst Biol. 2014 Jan 31;10(1):718. doi: 10.1002/msb.134708 (PMC4023404; doi:10.1002/msb.134708)
Supplement: Supplementary file 16 — Supplementary Table 2 [file MSB-10-1-718-s064.pdf]

Parameter	Definition	PS #1	PS #2	PS #3	PS #4	PS #5
k_1	Rate constant, MEK phosphorylation	0.919	0.786	26.9	12.9	61.9
k_{-1}	Rate constant, pMEK dephosphorylation	1.10	6.66	10.5	26.1	9.15
k_2	Rate constant, pMEK phosphorylation	2.32	44.8	18.9	7.44	18.2
k_{-2}	Rate constant, ppMEK dephosphorylation	0.186	0.176	0.377	0.375	0.448
K_i	Saturation constant, MEK kinase desensitization	5.25e3	4.81e3	5.93e3	4.70e3	8.46e3
s_{cT}	Total substrate in cytosol	3.25e-4	42.3	251	73.8	182
k_3	Rate constant, ERK phosphorylation, cytosol	0.468	0.302	2.01	2.09	1.43
k_{-3}	Rate constant, pERK dephosphorylation, cytosol	0.0286	0.0354	0.0824	0.0969	0.0712
k_4	Rate constant, pERK phosphorylation, cytosol	5.02	18.6	2.29e3	1.82e3	2.38e3
k_{-4}	Rate constant, ppERK dephosphorylation, cytosol	0.0431	3.99	132	37.6	105
k_n	Rate constant, ppERK nuclear import	4.93	1.90	1.18	2.86	0.801
k_{-n}	Rate constant, ERK nuclear export	0.909	1.10	0.541	6.23	2.21
$k_{on,c}$	Rate constant, ppERK-substrate association, cytosol	1.93e-4	0.0127	33.0	12.2	22.3
$k_{off,c}$	Rate constant, ppERK-substrate dissociation, cytosol	142	1.17	382	54.7	256
$k_{cat,c}$	Rate constant, substrate phosphorylation, cytosol	14.2	0.578	13.8	9.02	15.2
k_{-pc}	Rate constant, product dephosphorylation, cytosol	0.0275	3.34	2.20e-4	6.86e-4	1.75e-4
s_{nT}	Total substrate in nucleus	1.77e3	150	87.5	48.8	92.1
k_{-5}	Rate constant, pERK dephosphorylation, nucleus	0.0638	0.0367	0.402	3.94e-3	0.204
k_{-6}	Rate constant, ppERK dephosphorylation, nucleus	2.40	2.24	0.0450	0.794	0.0126
$k_{on,n}$	Rate constant, ppERK-substrate association, nucleus	6.05	6.97	3.52	178	26.8
$k_{off,n}$	Rate constant, ppERK-substrate dissociation, nucleus	0.0470	0.270	81.4	199	120
$k_{cat,n}$	Rate constant, substrate phosphorylation, nucleus	672	68.4	53.2	12.3	44.6
k_{-pn}	Rate constant, product dephosphorylation, nucleus	8.74e-4	1.83e-4	4.54e-3	0.0177	0.0193
α_M	Mono-/di-phosphorylated MEK in Western blot signal	0.0659	0.101	0.0242	9.21e-3	0.0157
α_E	Mono-/di-phosphorylated ERK in Western blot signal	0.0134	2.43e-3	0.522	0.124	0.513

Table S2. Selected parameter sets for the 'substrate' model. All parameters labeled as rate constants have units of min^{-1}; all others are dimensionless.
