# Supplementary material for: Data‐driven modeling reconciles kinetics of ERK phosphorylation, localization, and activity states
Source: Mol Syst Biol. 2014 Jan 31;10(1):718. doi: 10.1002/msb.134708 (PMC4023404; doi:10.1002/msb.134708)
Supplement: Supplementary file 17 — Supplementary Text [file MSB-10-1-718-s066.pdf]

Text S1: Modeling and Computational Methods Supplement

Data-driven modeling reconciles kinetics of ERK phosphorylation, localization, and activity states

Shoeb Ahmed, Kyle G. Grant, Laura E. Edwards, Anisur Rahman, Murat Cirit, Michael B. Goshe, and Jason M. Haugh

Kinetic model of ERK phosphorylation, nucleocytoplasmic shuttling, and interaction with substrates

We refer to the following kinetic model as the 'substrate' model, which was used to fit the data and generate predictions as described in the main text. We began with simple kinetic equations describing activation of MEK1/2, upstream of ERK1/2 phosphorylation. Owing to a nuclear export sequence, MEK is predominantly cytosolic, and so we only consider it to be localized in the cytosol. We consider three phosphorylation states: unphosphorylated (fraction m), mono-phosphorylated (fraction m_p), and diphosphorylated (fraction m_{pp}), and we assume that the total MEK expression is constant during the experimental time scale of interest. The rates of MEK phosphorylation are increased according to a dimensionless factor S, which represents the strength of the upstream input signal; we set $S = 1$ to correspond to signaling stimulated by 1 nM PDGF-BB, and we assume a step change at the time of growth factor addition; this minimal description of the network upstream of MEK is a simplification justified by previous work showing that the Ras- and PI3K-dependent inputs are saturated under these stimulation conditions (Wang et al, 2009; Cirit et al, 2010). Desensitization of the MEK activation response depends on negative feedback mediated by active ERK in the cytosol (c_{pp}). The final assumption is that the enzymes that phosphorylate and dephosphorylate MEK operate in the linear range, i.e. far from saturation. According to these assumptions, the differential equations in time t and associated initial conditions for the MEK species are as follows.

$$\frac{dm}{dt} = -\frac{k_1 S}{1+K_i c_{pp}} m + k_{-1} m_p; \quad m(0) = 1;$$

$$\frac{dm_p}{dt} = \frac{k_1 S}{1+K_i c_{pp}} m - k_{-1} m_p - \frac{dm_{pp}}{dt}; \quad m_p(0) = 0;$$

$$\frac{dm_{pp}}{dt} = \frac{k_2 S}{1+K_i c_{pp}} m_p - k_{-2} m_{pp}; \quad m_{pp}(0) = 0.$$

These equations invoke 5 adjustable rate constants: k_1, K_i, k_{-1}, k_2, and k_{-2}. Thus, this is the simplest model that incorporates double phosphorylation of MEK and its desensitization by active ERK; the goal here is to provide only as much detail as is necessary to fit the experimentally measured MEK phosphorylation response.

We next set our sights on the dynamics of ERK, starting with the cytosolic species. Following common modeling assumptions, the model considers that ERK is phosphorylated by MEK in the cytosol and must be diphosphorylated to i) translocate into the nucleus and ii) interact with cytosolic substrates. Phosphorylated ERK species are dephosphorylated by phosphatase(s) except when substrate-bound. Thus, cytosolic ERK is found in unphosphorylated (c), mono-phosphorylated (c_p), diphosphorylated (c_{pp}), and substrate-bound (cs) forms. It is assumed that export from the nucleus does not depend on phosphorylation status (Horgan & Stork, 2003); thus, export of unphosphorylated (n), mono-phosphorylated (n_p), and diphosphorylated (n_{pp}) ERK in the nucleus was assigned the same rate constant, k_{-n}. We confirmed that allowing different rate constants for export of the various

phospho-forms did not noticeably improve the quality of fit. Another simplifying assumption is to lump the many cytosolic substrates into one species (or class of species) that dominates the potential saturation of diphosphorylated ERK in the cytosol; the remaining substrates are assumed to possess far lower affinities for, and to equilibrate rapidly with, the pool of free, active ERK in the cytosol. The total amount of the dominant cytosolic ERK substrate — composed of the free substrate, the substrate in complex with ERK, and the phosphorylated product — is assumed to be constant (dimensionless s_{cT}). Defining the amount of the phospho-product as p_c, the amount of free, unphosphorylated substrate is given by $s_{cT} - cs - p_c$. According to these assumptions, and assuming that ERK phosphorylation by MEK and ERK dephosphorylation by phosphatases operate far from saturation, the conservation equations for the remaining cytosolic species in the model are as follows.

$$\frac{dc}{dt} = -k_3 m_{pp} c + k_{-3} c_p + k_{-n} n; \quad c(0) = 1;$$
$$\frac{dc_p}{dt} = k_3 m_{pp} c - k_{-3} c_p - k_4 m_{pp} c_p + k_{-4} c_{pp} + k_{-n} n_p; \quad c_p(0) = 0;$$
$$\frac{dc_{pp}}{dt} = k_4 m_{pp} c_p - k_{-4} c_{pp} - k_n c_{pp} + k_{-n} n_{pp} - \frac{dcs}{dt}; \quad c_{pp}(0) = 0;$$
$$\frac{dcs}{dt} = k_{on,c} c_{pp} \left(s_{cT} - cs - p_c \right) - \left(k_{off,c} + k_{cat,c} \right) cs; \quad cs(0) = 0;$$
$$\frac{dp_c}{dt} = k_{cat,c} cs - k_{-pc} p_c; \quad p_c(0) = 0.$$

There are 11 more adjustable parameters here: the rate constants k_3, k_{-3}, k_4, k_{-4}, k_n, k_{-n}, $k_{on,c}$, $k_{off,c}$, $k_{cat,c}$, and k_{-pc}, plus the aforementioned s_{cT}.

Rounding out the ERK dynamics, we describe the nuclear species. Here we follow the same assumptions and naming conventions as for the cytosolic species, with n in place of c. Nuclear ERK cannot be phosphorylated but is dephosphorylated at rates that are allowed to differ from those in the cytosol. Accordingly, the conservation equations for the corresponding nuclear species are as follows.

$$\frac{dn}{dt} = k_{-5} n_p - k_{-n} n; \quad n(0) = 0;$$
$$\frac{dn_p}{dt} = k_{-6} n_{pp} - k_{-5} n_p - k_{-n} n_p; \quad n_p(0) = 0;$$
$$\frac{dn_{pp}}{dt} = k_n c_{pp} - k_{-6} n_{pp} - k_{-n} n_{pp} - \frac{dns}{dt}; \quad n_{pp}(0) = 0;$$
$$\frac{dns}{dt} = k_{on,n} n_{pp} \left(s_{nT} - ns - p_n \right) - \left(k_{off,n} + k_{cat,n} \right) ns; \quad ns(0) = 0;$$
$$\frac{dp_n}{dt} = k_{cat,n} ns - k_{-pn} p_n; \quad p_n(0) = 0.$$

The 7 new parameters here are k_{-5}, k_{-6}, $k_{on,n}$, $k_{off,n}$, $k_{cat,n}$, k_{-pn}, and s_{nT}. All told, there are 25 adjustable parameters in this model: 23 kinetic parameters as outlined above plus 2 additional fitting parameters as outlined in the following section. We assert that this is at or close to the minimum number needed to capture the essential features of the pathway.

Hirashima formulated two kinetic models (a compartment model and a full model) that, like ours, include nucleocytoplasmic shuttling of ERK and ERK-substrate interactions (Hirashima, 2012). To achieve correspondence between the models, one would need to exclude certain processes, both from our model and from his. Hirashima's models do not include negative feedback, two-step phosphorylation and dephosphorylation of MEK or of ERK, phosphatase activity in the nucleus, or substrate dephosphorylation; in his compartment model, substrate is found only in the nucleus, and its free concentration is fixed, whereas his full model allows a single substrate to partition between the cytosol and nucleus and allows the system to reach steady state by including synthesis and degradation of the unphosphorylated substrate in the cytosol. Our model does not include (i.e., neglects) import of MEK or inactive ERK to the nucleus, binding of substrate to inactive ERK, or explicit accounting of the MEK-ERK complex.

Correspondence between model output and experimental data

The sum of the nuclear ERK species in our model, n_T, is fit to the data for nuclear translocation of fluorescent protein-tagged ERK and is defined as follows.

$$n_T = n + n_p + n_{pp} + ns\ .$$

Cytosolic and nuclear ERK kinase activity reporter (EKAR) data are assumed to be proportional to c_{pp} and n_{pp}, respectively. In other words, EKAR phosphorylation is assumed to be in rapid equilibrium with free, active ERK in each compartment, and EKAR dephosphorylation is also rapid; as explained in the main text, this assumption is justified by the data. For the biochemical data, mass spectrometry data for mono- and di-phosphorylated ERK (pERK and ppERK, respectively) correspond to the following quantities.

$$\text{pERK} = c_p + n_p;$$
$$\text{ppERK} = c_{pp} + cs + n_{pp} + ns.$$

Western blot data for phospho-MEK and phospho-ERK, are fit allowing for different affinities of the antibodies for the mono- and di-phosphorylated forms.

$$\text{phospho-MEK} = m_{pp} + \alpha_M m_p;$$
$$\text{phospho-ERK} = \text{ppERK} + \alpha_E \text{pERK}.$$

Thus, the fit introduces two additional adjustable parameters, α_M and α_E.

Definition of substrate buffering strengths

We define the ability of substrates to buffer diphosphorylated ERK as the concentration of substrate divided by the associated Michaelis constant, K_m. For cytosolic and nuclear substrates, respectively, these buffering strengths are defined as follows.

$$\frac{s_{c,T}}{K_{\mathrm{m},c}} = \frac{k_{on,c} s_{cT}}{k_{off,c} + k_{cat,c}};\quad \frac{s_{n,T}}{K_{\mathrm{m},n}} = \frac{k_{on,n} s_{nT}}{k_{off,n} + k_{cat,n}}\ .$$

Alternative model topologies

Three other models, listed below, were also evaluated. These differ from the regular 'substrate' model as follows.

1) The 'no substrate' model, which is also referred to as the core model in the main text. This is a simplified model in which interactions with substrates are absent ($cs = ns = 0$). This model has 17 fit parameters, 8 fewer than the regular 'substrate' model, since s_{cT}, $k_{on,c}$, $k_{off,c}$, $k_{cat,c}$, s_{nT}, $k_{on,n}$, $k_{off,n}$, and $k_{cat,n}$ are not needed.

2) The ‘no substrate/importin’ model. Here we neglected substrate interactions as in the ‘no substrate’ model but allowed for a two-step nuclear import of diphosphorylated ERK. A new species *ni*, roughly corresponding to di-phosphorylated ERK in complex with importin, is introduced. Its differential equation and the modified differential equation for n_{pp} are as follows.

$$\frac{dni}{dt} = k_n c_{pp} - k_{imp} ni; \quad ni(0) = 0;$$

$$\frac{dn_{pp}}{dt} = k_{imp} ni - k_{-6} n_{pp} - k_{-n} n_{pp}; \quad n_{pp}(0) = 0.$$

Relative to the ‘no substrate’ model, the importin model has the additional parameter k_{imp}, and therefore it has 18 fit parameters (7 fewer than the ‘substrate’ model). In the fit to experimental data, *ni* is included in the quantities n_T and ppERK.

3) The ‘substrate/importin’ model. This model accounts for both substrate interactions and the two-step nuclear import of ERK. With the inclusion of k_{imp}, this model variation has 26 fit parameters, one more than the ‘substrate’ model.

Parameter fitting algorithm

Model fitting/alignment to our data set was carried out using a Monte Carlo routine based on the Metropolis algorithm (Metropolis et al, 1953). The algorithm was implemented in MATLAB (MathWorks), adapted from a routine described in detail previously (Cirit & Haugh, 2012). To set all of the data on a similar scale, the data for each time course was normalized again to set the mean value equal to unity. In brief, the algorithm works as follows.

1) An initial set of parameters is chosen.

2) The dimensionless model output is computed using the stiff solver *ode15s*.

3) The model outputs based on the current parameter set *i* are multiplied by alignment factors to best align the model with the normalized data (which are in arbitrary units). The value of each alignment factor a_{ij}, one for each of the 9 time courses *j*, is chosen such that the sum of squared deviations (SSD) comparing measured and calculated values is minimized. The minimum SSD_{ij} was found by systematically subdividing the range of possible a_{ij} values until SSD_{ij} could no longer be reduced by more than 0.1%. The minimum SSD_{ij} values thus obtained are saved and used to evaluate the closeness of fit, as described below.

4) A new set of parameters is determined from the old set as follows.

$$k_{i+1} = k_i\left(1 + \alpha\,\text{randn}\right).$$

where k_i is one of the model parameters for iteration *i*, randn is a random number drawn for each parameter from a standard normal distribution, and α is a parameter of the algorithm that governs how much the parameter values tend to change between iterations. After experimentation we chose to use a value of $\alpha = 0.03$ throughout the analysis reported here. If any of the new parameters is below 10^{-4} or greater than 10^{4}, the new value is thrown out, and another value is drawn based on the old value.

5) Steps 2-4 are repeated using the new parameter set, and its SSD_{ij} are evaluated. A cumulative SSD, $cSSD_i$, is calculated as a weighted sum of the SSD_{ij} as follows.

$$cSSD_i = \sum_j w_j SSD_{ij}$$

After experimentation we chose the following values for the weighting factors, w_j. To emphasize the 3 core imaging experiments (nuclear translocation and the two EKAR readouts), we assigned the highest weighting factor, $w = 10$. A weighting of $w = 5$ was used for the 4 biochemical experiments (immunoblotting and mass spectrometry data), and $w = 1$ was used for the 2 EKAR experiments with MEK inhibition imposed after stimulation.

6) The value of $cSSD_i$ is compared to that of the previous iteration. If it is less (better fit), the parameter set is accepted. If it is greater, it is accepted with a probability given by the following formula.

$$p_{i+1} = \exp\left[-\frac{\left(cSSD_{i+1} - cSSD_i\right)}{T_i}\right];$$

$$T_i = \beta cSSD_i.$$

A value of the algorithm parameter β equal to 0.01 was used. If the new parameter set is not accepted by these criteria, it is thrown out, and the procedure is repeated using the previous parameter set as the input.

7) The procedure is repeated until the desired number of accepted parameter sets (at least 50,000) is achieved. All of the accepted parameter sets are saved in a matrix for further analysis.

8) The ensemble was culled to determine the 10,000 'best' parameter sets as follows. First, we imposed certain cut-offs for the values of SSD_{ij}. For each of the 3 core imaging experiments, a maximum SSD_{ij} value of 0.75 was chosen. For each of the other 6 experiments, a maximum SSD_{ij} value of 2.0 was used. Then, we ranked the remaining parameter sets by $cSSD_i$ value and selected the 10,000 with the lowest values.

Statistics for this ensemble for the 'substrate' model are summarized in **Table S1** below. The same information is also shown in box plot form, with the parameters split into two groups according to the ratio of the first and third quartiles (height of the box on a log scale), but otherwise ordered by appearance in Table S1. Those parameters with smaller ratios are considered to be better constrained by the fit to the data (although in the case of the parameter K_i, it is assigned arbitrarily large values, close to the maximum).

Ensemble averaging and analysis
With the ensemble of parameter sets (along with their alignment factors) saved as a matrix, MATLAB was used to recalculate the aligned model output for each parameter set and store those values in a larger matrix. For each experimental condition and time point, an ensemble mean and standard deviation (n = 10,000) were computed, and these values were used to compare the model with the experimental data.

Selected parameter sets
As explained in the main text, we selected five parameter sets from the ensemble based on their substrate buffering strengths in the cytosol and nucleus (as shown in Fig. 5). Their parameter values are listed in **Table S2** below.
